# Plummeting anthropogenic environmental degradation by amending nutrient-N input method in saffron growing soils of north-west Himalayas

**DOI:** 10.1038/s41598-021-81739-x

**Published:** 2021-01-28

**Authors:** Anil Sharma, Sarvendra Kumar, Shakeel Ahmad Khan, Amit Kumar, Javid Iqbal Mir, Om Chand Sharma, Desh Beer Singh, Sanjay Arora

**Affiliations:** 1 ICAR-Central Institute of Temperate Horticulture, Old Air Field, KD Farm, Rangreth, Jammu and Kashmir 191132 India; 2 ICAR- Indian Agriculture Research Institute, New Delhi, India; 3 ICAR-Central Soil Salinity Research Institute, Regional Research Station, Lucknow, UP 226002 India

**Keywords:** Environmental social sciences, Sustainability

## Abstract

Nitrous-oxide emission and nitrate addition from agriculture to earth’s environment are two main agriculture related anthropogenic causes of environmental degradation that needs greater attention. For addressing the aforesaid issue, new techniques/practices need to be developed and implemented. The present investigation, which was focused on this issue, resulted in developing a new mode of nitrogen (N) placement, i.e. ‘mid rib placement upper to corms in two splits (MRPU-2S)’, that could reduce nitrous oxide N emission by around 70.11% and, nitrate N leaching and runoff by around 68.26 and 67.09%, respectively, over conventional method, in saffron growing soils of northwest Himalayas. Besides plummeting environmental degradation, MRPU-2S further resulted in enhancing saffron yield by 33.33% over conventional method. The findings of the present investigation were used to develop new empirical models for predicting saffron yield, nitrate N leaching and nitrous-oxide N emission. The threshold limits of nitrate N leaching and nitrous oxide N emission have also been reported exclusively in the present study.

## Introduction

Pessimistic modification in the intrinsic composition of nature, by various human induced activities and practices, results in anthropogenic environmental degradation. This occurs when the earth’s natural resources are depleted, due to human interference, and the environment is compromised in the form of pollution in the soil, water and air. Along with industries and transportation, agriculture is considered as one of the major causes of pollution that can corrupt environment considerably. Fertilizers, especially nitrogenous fertilizers, and pesticides are known to be the major culprits, amongst agricultural inputs, responsible for such pollution. Every year, about 67.84 million tons of nitrogen is applied to agricultural lands worldwide^1^. Most of this nitrogen escapes the agricultural system^2^ and become pollutants^3^. Nitrogen when applied in an agricultural field encounters five major processes viz. taken up by crop, fixes in soil as reserve, leaches from the root zone, escapes to atmosphere as gas and moves as surface runoff^2,4,5^. Later three of these five processes are considered as the main contributor to environmental deterioration. These processes are responsible for nitrate pollution, in ground and surface water bodies, and nitrous oxide emission (N_2_O) which is one of the greenhouse gases (GHGs) responsible for global warming.

Contamination of surface and ground water by leached nitrate from agricultural fields is a global phenomenon that has impelled social and political pressure to trim down nitrate leaching leading to contamination of water^6^ and consequent environmental degradation. As per global estimates^7^ around 50% of the total nitrogen (N) lost (that constitutes approximately 10–20% of applied N)^8^ of the nitrogen applied to the agricultural fields is lost by leaching^9,10^, and becomes a part of ground water. About 50% of the world’s drinking water^11^ and 40% of agricultural irrigation water^12^ is supplied by ground water. The ground water resources also maintain surface water bodies like lakes, rivers and wetlands^13^. So, leached as well as surface runoff nitrate N is diffused to these water resources and poses threat to ground as well as surface water quality globally^14,15^. The Environmental Protection Agency (EPA) has since adopted the 10 mg L^−1^ standard as the maximum contaminant/permissible level (MCL) for nitrate–nitrogen in water. Higher concentrations than the MCL can cause severe public health risk^16,17^, which at present is a big global concern.

Nitrogen fertilization in agricultural soils is not merely accountable for nitrate pollution, but also for the greatest amount of nitrous oxide (N_2_O) emissions among all the anthropogenic sources^18,19^. Agriculture is a major contributor^20,21^ that accounts for about 60% of N_2_O emissions worldwide^22^ and inefficient use of externally added N, by crops, is considered responsible for this emission^23^. Nitrous oxide emission during the last few decades has increased and its levels in the atmosphere have amplified, which is not good, as it contributes to global temperature change^2^, crop pest infestation^24^ and human health risks^25^.

The aforesaid nitrogen losses, in agricultural crops, and their pollution potential has persuaded researchers to identify easily adaptable management practices that ensure enhanced N use efficiency and reduction in its losses that cause environmental deterioration. Amongst these agricultural crops N management of Saffron (*Crocus sativus*), one of the most expensive agricultural crops of the world^26^, as its approximate price ranges from US$500 to US$5,000 per pound, needs greater consideration. India is the second largest producer of Saffron after Iran^27^ and in India it is grown only in cold humid regions of North West Himalayas. In the last few decades a decrease in area and productivity of this crop has been noticed. Along with others, declining soil fertility^27^ and inefficient nutrient, especially N, management are deemed as the factors responsible for this decline. The major problem in the current mode of N application practice (broadcasting/surface application) is its low nutrient N use efficiency as a substantial amount of nitrogen gets wasted as N losses^7^. Low nitrogen use efficiency results in low biomass production, whole of which is incorporated in the soil, which further leads to low soil organic carbon stocks and hence low saffron yield in sequential years. Besides the said mode leaves a substantial amount of fertilizer for weeds. Further, there is a need of rainfall or assured irrigation (in case of broad casting) to move N into the root zone otherwise nutrient remain on soil surface making it unavailable to the plant root system. So for efficient N management, right placement of nutrient in the soil is a key component for escalating plant utilization and curtailing nutrient N losses responsible for environmental degradation. Even if not in all but in few of the important agricultural crops grown worldwide, the right nutrient N placement mode is standardized and followed, then it will help not only in enhancing use efficiency of external N supplied but will reduce environmental degradation due to N too.

Keeping in view the above-mentioned facts, it becomes obligatory to alleviate nitrogen losses leading to environmental/ecological degradation. This can be done by introducing agricultural practices that enhance N use efficiency of crop plants^28–30^ and right mode of N application/placement and its rate are a few of such options. But as rate of N application for the crop has already been standardized/recommended, so the present investigation was aimed to standardize/evaluate input method/mode of application of the recommended dose of nutrient N, with the hypothesis that mode of application can be the reason and may have a significant influence on N losses and use efficiency. So, in present investigation, for the very first time, the effect of various modes of nitrogen applications/placement, conventional as well as introduced/amended, was investigated and their impacts on environment i.e. nitrate pollution, nitrous oxide emission and saffron production was revealed. Although the present investigation was carried out in saffron growing soils only, but the information/recommendation generated from it can be applied in other soils too.

## Results

### Nitrogen losses, as nitrate leaching and surface runoff, and its pollution potential

**Figure 1:**
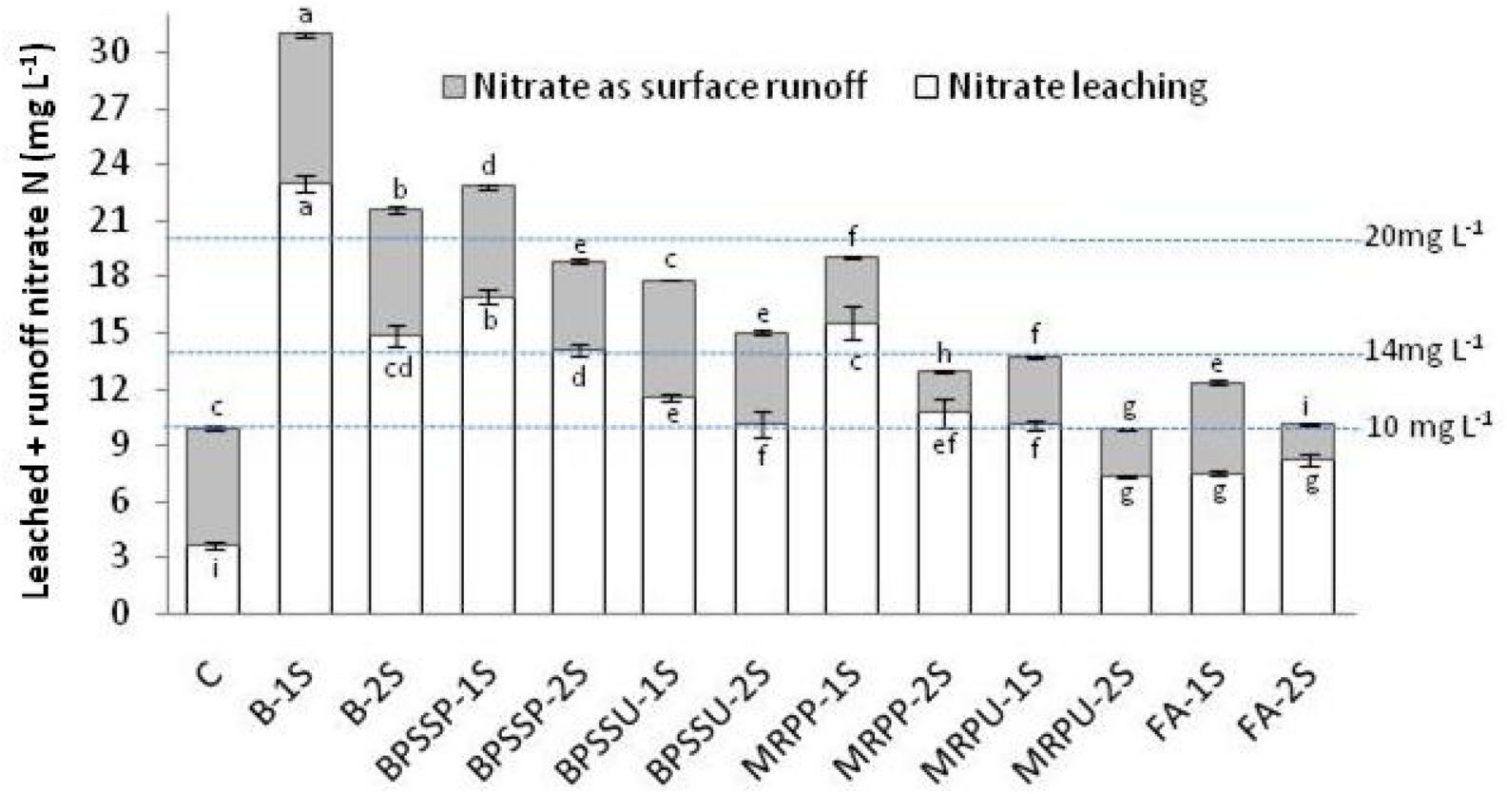
Nitrogen losses (as leaching and surface run off) and their cumulative pollution potential, as effected by various N input methods in saffron growing cold humid region of North West Himalayas (Maximum permissible limit (MPL), as per Environmental Protection Agency (EPA) guidelines, of Nitrate N in ground water/water bodies = 10 mg L^−1^; Negligible polluting potential (≤ 10 mg L^−1^); Low polluting potential (> 10 ≤ 14 mg L^−1^); High polluting potential (> 14 ≤ 20 mg L^−1^); Very high polluting potential (> 20 mg L^−1^)).

Influence of various nutrient-N input methods on its losses in the form of nitrate, leading to environmental pollution was revealed (Fig. 1). Nitrate N leaching was well below the maximum permissible limit (MPL) of 10 mg L^−1^, in four treatments reflecting their negligible pollution potential due to nitrate N leaching. Least nitrate N leaching (3.6 mg L^−1^) was noticed in the control where as in other three treatments, i.e. MRPU-2S, FA-1S and FA-2S, nitrate N leaching amounted to 7.3, 7.5 and 8.2 mg L^−1^, which were statistically at par with each other. Nitrate N leaching in conventional method 1 (BS-1S) was 23 mg L^−1^ and was well above the MPL, thus leading to very high polluting potential (> 20 mg L^−1^). In ‘high polluting potential category’ (> 14 ≤ 20 mg L^−1^), the other treatments were B-2S (conventional method II), MRPP-1S and BPSSP-1S with values of nitrate N leaching being 14.8, 15.5 and 16.9 mg L^−1^, respectively. Nitrate N leaching in remaining treatments was in ‘low pollution potential category’ (> 10 ≤ 14 mg L^−1^).

So far as nitrate N addition through surface runoff is concerned, it ranged between 1.9 to 7.9 mg L^−1^. As ground water also contributes to surface water bodies and vice-versa, so cumulative nitrate N addition, due to leaching and surface runoff was also studied (Fig. 1). Cumulatively, other than control (9.9 mg L^−1^ nitrate N leaching) only one treatment MRPU-2S was below MPL with value being 9.9 mg L^−1^, thus having negligible pollution potential. Both the conventional methods were cumulatively in very high polluting potential category with values being 30.9 and 21.6 mg L^−1^.

### Nitrogen losses, as nitrous oxide emission, and its global warming potential (GWP)

**Figure 2:**
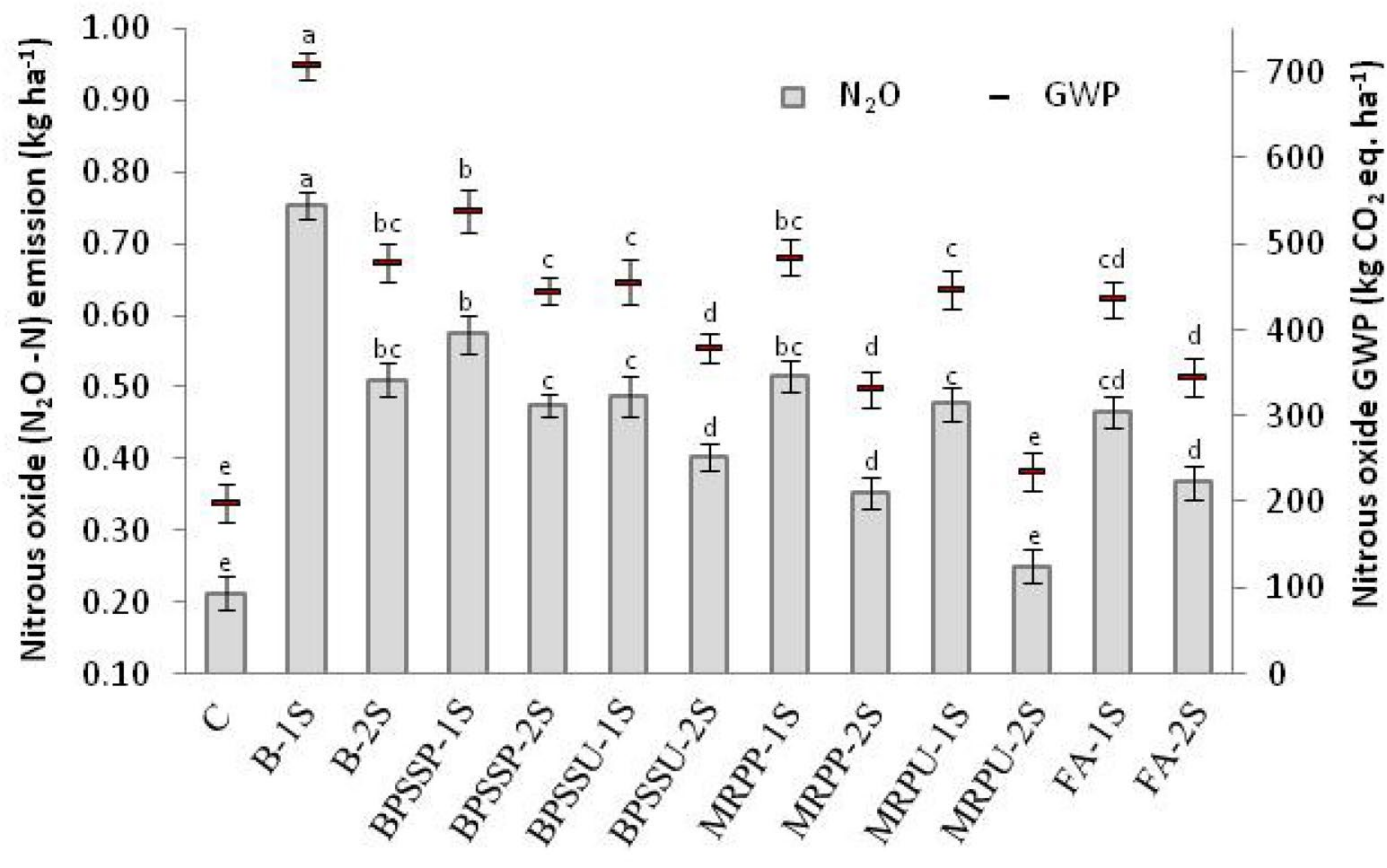
Nitrogen loss (as N_2_O–N) and its global warming potential as effected by various N input methods in saffron growing soils of cold humid region of North West Himalayas.

A significant influence of various N input methods on N_2_O–N emission and consequently global warming potential (GWP) was observed (Fig. 2). Highest N_2_O–N emission amounting to around 0.75 kg ha^−1^ was noticed in B-1S (conventional I) treatment followed by BPSSP-1S treatment (0.57 kg ha^−1^) which was at par with MRPP-1S and BS-2S (conventional method II) with emission values for the later two being 0.52 and 0.51 kg ha^−1^, respectively. Least emission, which was statistically as low as in control (0.21 kg ha^−1^) was observed in MRPU-2S that amounted to around 0.25 kg ha^−1^. The global warming potential of N_2_O also followed similar trends (statistically also). Highest GWP was observed in B-1S (706 kg CO_2_ eq. ha^−1^) and least GWP due to N_2_O emission was observed in MRPU-2S (234.90 kg CO_2_ eq. ha^−1^) which was at par with control (200.06 kg CO_2_ eq. ha^−1^).

### Nitrogen use and accumulation efficiency

Nitrogen use (NUE) and accumulation (NAE) efficiency also got affected by various N input methods (Table 1). Highest nitrogen use and accumulation efficiency were observed in MRPU-2S treatment which was to the extent of 23.9 and 33.7%, respectively, and was statistically significant over other treatments. MRPP-2S and MRPU-1S were next with values for NUE being 21.6 and 21.0% and NAE being 31.4 and 30.8%.

**Table 1:** NUE, NAE, yield, crocin and safranal content as affected by various N input methods in saffron growing cold humid region of North West Himalayas.

Treatment	Yield (kg ha^−1^)	Crocin (mg g^−1^)	Safranal ( mg g^−1^)	NUE (%)	NAE (%)
C	1.16 (± 0.006)^g^	16.71 (± 0.074)^g^	0.14 (± 0.015)^c^	**–**	**–**
B-1S	1.44 (± 0.003)^ef^	25.57 (± 0.089)^b^	0.29 (± 0.015)^a^	12.8 (± 0.76)^e^	22.6 (± 0.46)^e^
B-2S	1.45(± 0.064)^e^	18.36 (± 0.058)^f^	0.16 (± 0.012)^c^	16.5 (± 0.04)^d^	26.3 (± 0.39)^d^
BPSSP-1S	1.38 (± 0.043)^f^	12.43 (± 0.072)^i^	0.21 (± 0.012)^b^	15.9 (± 0.34)^d^	25.7 (± 0.06)^d^
BPSSP-2S	1.42 (± 0.006)^ef^	21.86 (± 0.048)^cd^	0.19 (± 0.015)^bc^	17.4 (± 0.95)^d^	27.2 (± 0.88)^d^
BPSSU-1S	1.61(± 0.012)^c^	16.70 (± 0.015)^g^	0.13 (± 0.009)^c^	18.8 (± 1.13)^cd^	28.6 (± 0.78)^cd^
BPSSU-2S	1.62 (± 0.003)^c^	21.50 (± 0.030)^d^	0.16 (± 0.010)^c^	19.7 (± 0.33)^c^	29.4 (± 0.29)^c^
MRPP-1S	1.78 (± 0.012)^b^	10.79 (± 0.184)^j^	0.29 (± 0.018)^a^	17.2 (± 0.71)^d^	27.0 (± 0.67)^d^
MRPP-2S	1.80 (± 0.007)^b^	19.21 (± 0.110)^e^	0.22 (± 0.015)^b^	21.0 (± 0.20)^b^	30.8 (± 0.80)^b^
MRPU-1S	1.83 (± 0.006)^b^	17.00 (± 0.127)^g^	0.20 (± 0.015)^bc^	21.6 (± 0.26)^b^	31.4 (± 0.63)^b^
MRPU-2S	1.92 (± 0.012)^a^	26.64 (± 0.039)^a^	0.30 (± 0.012)^a^	23.9 (± 1.01)^a^	33.7 (± 0.74)^a^
FA-1S	1.53 (± 0.006)^d^	22.00 (± 0.381)^c^	0.28 (± 0.015)^a^	18.3 (± 0.11)^cd^	28.1 (± 0.45)^cd^
FA-2S	1.57 (± 0.006)^cd^	14.57 (± 0.170)^h^	0.19 (± 0.015)^bc^	17.2 (± 0.19)^d^	27.0 (± 0.09)^d^

### Relationship of nitrogen losses with NUE and NAE

**Figure 3:**
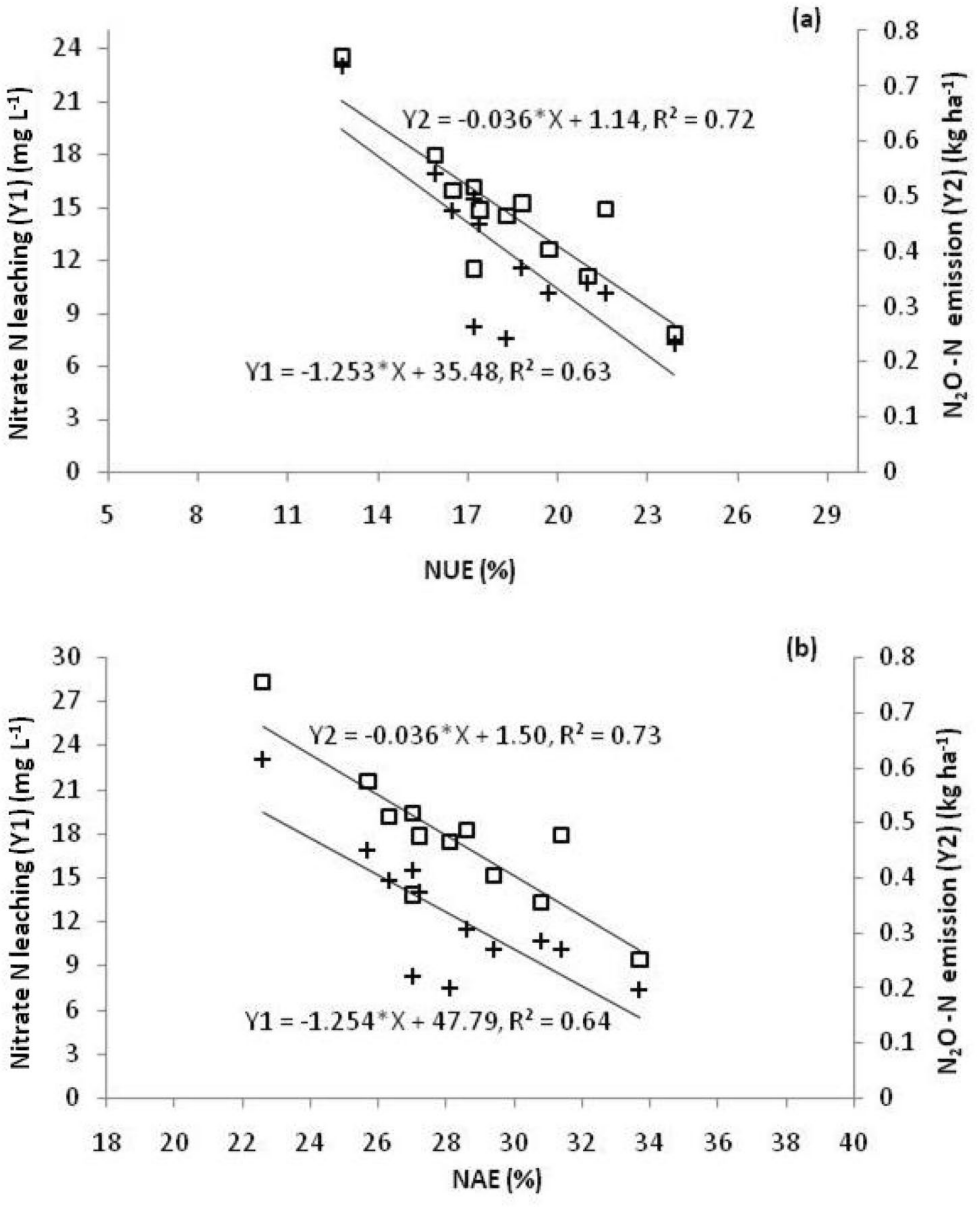
Relationship between Nitrogen losses and (**a**) nutrient use and (**b**) nutrient accumulation efficiency in saffron growing cold humid region of North West Himalayas.

A linear, but negative relation of NUE and NAE with Nitrate N leaching as well as Nitrous oxide emission was observed (Fig. 3). On the basis of these relationships empirical models to calculate and quantify nitrate leaching and nitrous oxide emission, from relatively easily determinable NUE and NAE, were developed which are presented as Eqs. (1)–(4).

On the basis NUE

1 $${\text{Nitrate N leaching }}\left( {{\text{mg }}{{\text{L}}^{ - 1}}} \right) \, = \, - 1.253*{\text{NUE }} + 35.48,$$

2 $${{\text{N}}_2}{\text{O}} - {\text{N emission }}\left( {{\text{kg h}}{{\text{a}}^{ - 1}}} \right) \, = \, - 0.036*{\text{NUE }} + 1.14.$$

On the basis NAE

3 $${\text{Nitrate N leaching }}\left( {{\text{mg }}{{\text{L}}^{ - 1}}} \right) \, = \, - 1.254*{\text{NAE }} + 47.79,$$

4 $${{\text{N}}_2}{\text{O}} - {\text{N emission }}\left( {{\text{kg h}}{{\text{a}}^{ - 1}}} \right) \, = \, - 0.036*{\text{NAE }} + 1.50.$$

Correlation analysis was performed to understand the relationship between NUE and N losses. Results revealed that NUE was negatively and significantly related to gaseous (r = − 0.852**) and leaching (r = − 0.798**) N losses, however no significant relationship of NUE with N losses as surface runoff was observed.

### Saffron yield and apocarotenoid contents

Various N input methods affected saffron yield and its apocarotenoid contents too (Table 1). A significantly higher yield of 1.92 kg ha^−1^ was observed in MRPU-2S treatment. Treatments MRPU-1S, MRPP-2S and MRPP-1S were next with a yield of 1.83, 1.80 and 1.78 kg ha^−1^, respectively. Least yield was noticed in BPSSP-1S (1.38 kg ha^−1^) which was at par with B-1S (conventional method I) (1.44 kg ha^−1^) and BPSSP-2S (1.42 kg ha^−1^).

The results also showed a significant variation among various N input methods with respect to apocarotenoid contents (Table 1). MRPU-2S had the highest total concentration of both crocin (26.64 mg g^−1^ of stigmas) and safranal (0.30 mg g^−1^).

### Saffron yield as influenced by nitrogen losses

**Figure 4:**
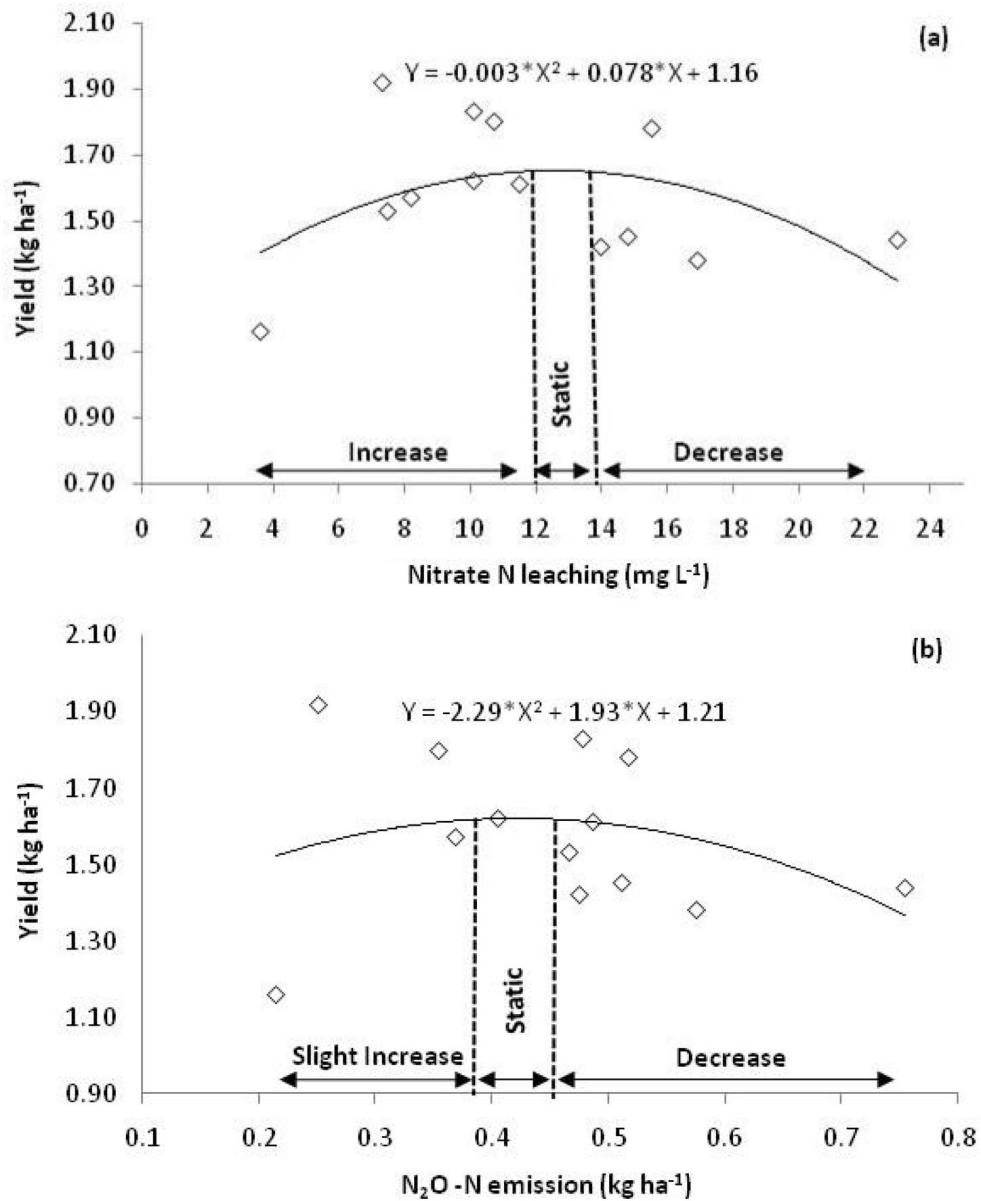
Effect of Nitrogen losses, (**a**) Nitrate leaching (**b**) N_2_O–N emission, on saffron yield in saffron growing cold humid region of North West Himalayas.

Besides degrading environment, N losses affected saffron yield too (Fig. 4). It was observed that during the initial losses of N, whether through nitrate N leaching or N_2_O–N emission, yield showed a slight increase, and then static and decreased thereafter.

It was observed that up to 12 mg L^−1^ nitrate leaching yield showed a slight increase, then remained somewhat static up to 13.9 mg L^−1^ and after that decreased. It means if nitrate leaching exceeds 13.9 mg L^−1^, a decrease in yield starts and its extent can be quantified by a polynomial empirical model given as Eq. (5). Almost similar trends in saffron yield in relation to N_2_O–N emission were also observed. It was revealed that yield increased up to 0.38 kg ha^−1^ N_2_O–N emission, remained static between 0.38 to 0.45 kg ha^−1^ and decreased thereafter. This further suggests that when N_2_O–N emission goes above 0.45 kg ha^−1^ it affects saffron yield negatively. The extent of the decrease can be quantified from the polynomial empirical model presented as Eq. (6).

5 $$\begin{gathered} {\text{Saffron yield }}\left( {{\text{kg h}}{{\text{a}}^{ - 1}}} \right) \, = \, - 0.003*{\left( {{\text{N}}{{\text{O}}_3}^- {\text{leaching in mg }}{{\text{L}}^{ - 1}}} \right)^2} \hfill \\ + 0.078*\left( {{\text{N}}{{\text{O}}_3}^- {\text{leaching in mg }}{{\text{L}}^{ - 1}}} \right) \, + \, 1.16 \hfill \\ \end{gathered}$$

6 $$\begin{gathered} {\text{Saffron yield }}\left( {{\text{kg h}}{{\text{a}}^{ - 1}}} \right) \, = \, - 2.29*{\left( {{{\text{N}}_2}{\text{O}} - {\text{N emission kg h}}{{\text{a}}^{ - 1}}} \right)^2} \hfill \\ + 1.93*\left( {{{\text{N}}_2}{\text{O}} - {\text{N emission kg h}}{{\text{a}}^{ - 1}}} \right) \, + \, 1.21 \hfill \\ \end{gathered}$$

The threshold values of Nitrate leaching (13.9 mg L^−1^) and Nitrous oxide emission (0.45 kg ha^−1^ N_2_O–N) after which yield starts decreasing have been reported exclusively in the present study.

### Change in nitrogen losses, leading to environmental degradation, and saffron yield in comparison to conventional N input methods

**Figure 5:**
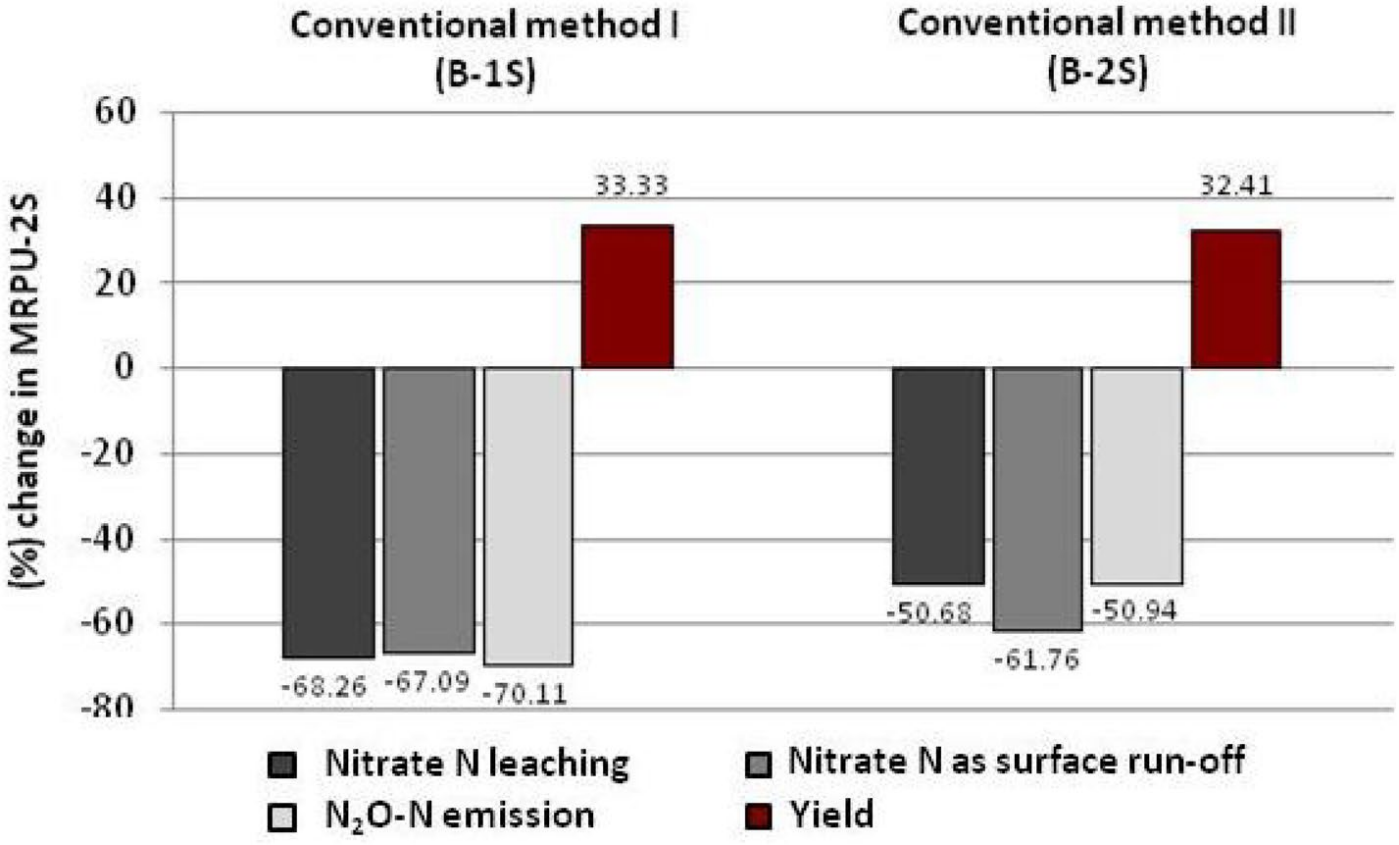
Per-cent change in nitrogen losses leading to environmental pollution, and saffron yield in MRPU-2S method over conventional (practiced) method in saffron growing cold humid region of North West Himalayas.

Data analysis revealed that N application through MRPU-2S resulted in significant reduction in N losses (Fig. 5). In the aforesaid treatment nitrate leaching, nitrate as surface runoff and nitrous oxide N emission reduced by 68.26, 67.09 and 70.11% over conventional method I (B-1S) that is practiced by most of the saffron growers. In case of conventional method II i.e. B-2S, these N losses reduced by 50.68% (as nitrate leaching), 61.76% (as surface runoff) and 50.94% (as nitrous oxide N emission) in MRPU-2S treatment. Impact of N input through MRPU-2S on saffron yield was also determined and it was observed that yield in this treatment increased over both the conventional methods to an extent of 33.33 and 32.41% in conventional method I (B-1S) and conventional method II (B-2S), respectively.

### Clay minerals and nitrate leaching

**Figure 6:**
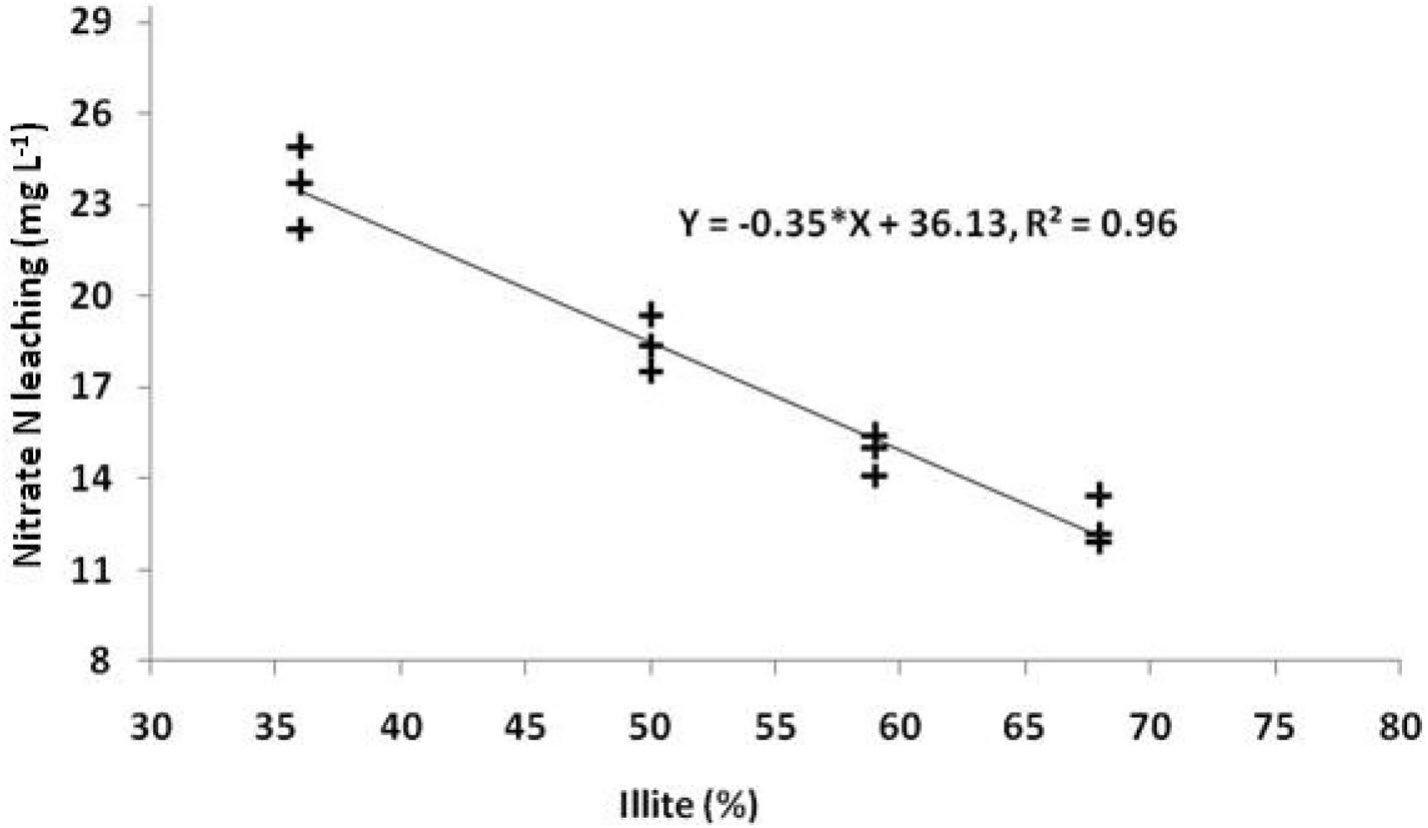
Nitrate N leaching as influenced by illite content in saffron growing cold humid region of North West Himalayas.

Soils from four locations, representative of major saffron growing areas of cold humid south western Himalayas were studied for clay mineralogy with the hypothesis that clay minerals do have their influence on nitrate leaching from soils. The X-ray diffractograms of the soil clays clearly exhibited the dominance of illite in these soils. After analyzing the relationship of nitrate leaching with illitic clay minerals (Fig. 6), a linear but negative relation was observed which can be understood from the Eq. (7).

7 $${\text{Nitrate leaching}}\left( {{\text{mg }}{{\text{L}}^{ - 1}}} \right) \, = \, - 0.35*{\text{Illite }}\left( \% \right) \, + \, 36.13.$$

## Discussion

Cumulatively low nitrate addition and hence negligible pollution potential in MRPU-2S can be attributed to the correct placement of N fertilizer, as correct placement often improves the efficiency by which plants take up nutrients and thus can protect both surface and ground water quality^31^. Rib or band placement reduces potential erosion losses and thus reduces surface runoff. More surface losses can also be attributed to low to medium infiltration rate (16 mm h^−1^) of these soils. More leaching losses in conventional methods may be because of the high nitrification rate followed by the oxidation of the nitrite to nitrate in these treatments. It was further observed that when N was applied in two splits the losses were less which means that N input was meeting plant demand as was applied at critical stages^32^. It was further established that when N was placed midrib upper to the corms it was efficiently taken up by plants as in single sided placement and parallel placement a significant portion of it could not be taken up by plants and got removed as leaching (as a significant part of nitrogen moves to offside from where the roots are relatively far).

Low N_2_O–N emission in MRPU-2S can be attributed to decrease in volatilization losses of fertilizer N due to its incorporation in soil^31^. When fertilizer N is placed closer to the roots, the plant is likely able to use the nutrient more efficiently, especially at earlier stages when plant roots are small and localized^33^. More N_2_O–N emission means more concentration of this gas in the atmosphere and hence more contribution to the greenhouse effect and consequently more global warming potential.

Localized placement of N in the root zone, in MRPU-2S, resulted in maximum uptake of nitrogen^34^ that further resulted in high NUE and NAE in this treatment. While in conventional and other methods, although the extent was varied, the N losses were more and hence less uptake resulted in low NUE. Moreover deep placement of N fertilizer saves N fertilizer and increase NUE^35^. Low NUE because of inappropriate applications of nitrogen (N) fertilizer have also been noticed by other researchers too^36,37^. Inappropriate N fertilization results in great N losses, and thus environmental problems, such as groundwater and surface water contamination, greenhouse gas increases, and soil quality degradation^19,38^.

Further a linear but negative relation of NUE and NAE with both nitrate N leaching and nitrous oxide N emission can be because of the reason that as N losses increases, less nitrogen retains in the soil for plant roots and thus plants cannot optimize N utilization because of a lesser amount of uptake and accumulation.

More saffron yield in MRPU-2S can be attributed to more NUE and reduced nitrogen losses of this treatment when compared to others. In existing N application practice (broadcasting) there exists three main disadvantages viz. underutilization of N by plant roots as it moves laterally over long distance, weed growth gets stimulated and N gets fixed due to its exposer to large masses of soil. With correct N management, in present case ‘N application’, saffron can produce well^39^. So far no such study has been attempted in saffron crop but somewhat similar results have been reported in a previous study in Canada in wheat crop, where wheat yield was greater with subsurface N treatments than with surface N broadcast^40,41^. Researchers also reported that wheat grain yield after broadcast application was significantly lower than with deep banding of N fertilizer^42^. Uptake of N by weeds in surface broadcast of basal fertilizer may result in low yields^43^ in conventional methods. More amount of apocarotenoid in MRPU-2S treatment, compared to others can again be attributed to high N uptake as N plays its role in metabolism.

A polynomial relationship of yield with N losses was observed. A slight initial increase in saffron yield with increasing N losses can be attributed to the high rate of initial N mineralization that results in temporary high uptake and decrease thereafter. A few regression models for estimating yield have already been given by^44,45^, but most of these models are based on other parameters like weather or crop growth parameters. The N management practice suggested in the present investigation will influence NUE and consequently N losses and ultimately yield. So these regression models can be of great significance for saffron yield anticipation.

Observance of lower N losses in MRPU-2S in comparison to conventional method can be due to N fertilizer input/placement at deeper soil depths, in MRPU-2S, that have relatively low soil temperature and total organic carbon levels compared to the soil surface and shallow depths, and thus expose fertilizer to reduced soil microbial activity and hence reduce soil N_2_O–N emissions and nitrate N leaching^46^. An increase in yield in MRPU-2S can be attributed to high NUE of the said treatment. These findings hold up our assumption that by escalating saffron yields through amending N input method, the negative ecological impacts associated with saffron crop can also be reduced and less N will be available for losses.

The illitic nature of saffron growing soils could be attributed to micaceous parent material of these soils^47^. Further a linear but negative relationship of illite with nitrate N leaching was noticed. This could be attributed to immobilization of nitrogen as NH_4_^+^ ion, as it displaces K^+^ ion from diurinal cavities of illitic clay minerals because of somewhat close ionic radii^48,49^, especially in illitic/micaceous clay minerals. This further results in a reduced nitrification rate followed by a decrease in oxidation of the nitrite to nitrate and hence reduced leaching of nitrate.

## Conclusions

Growers cannot control natural factors like soil properties and climatic conditions, that affect N losses, but anthropogenic N losses leading to environmental degradation can be reduced by efficient nutrient management practices. Mode of N fertilizer placement is one of these. After scrutinizing the results of present investigation, it can be concluded that nitrogen placement in mid rib upper to corms in two splits, resulted in reduced N losses responsible for environmental pollution and subsequently enhanced yield too. Besides, the soils with more illitic clay minerals encountered less N losses due to leaching, which mean illite dominating soils should be encouraged for saffron cultivation in cold humid regions of northwest Himalayas. Empirical models developed will be of great significance for researchers, planners and policy makers as these can predict and quantify yield as well as N losses. Further, the researchers working in related field, especially in developing countries, will also have to consider the fact that instead of going to expensive mitigation options lets us first amend, consolidate and extend the easily controllable management practices that can reduce N losses (as leaching, surface runoff or as nitrous oxide emission) responsible for ecological degradation.

## Materials and methods

**Figure 7:**
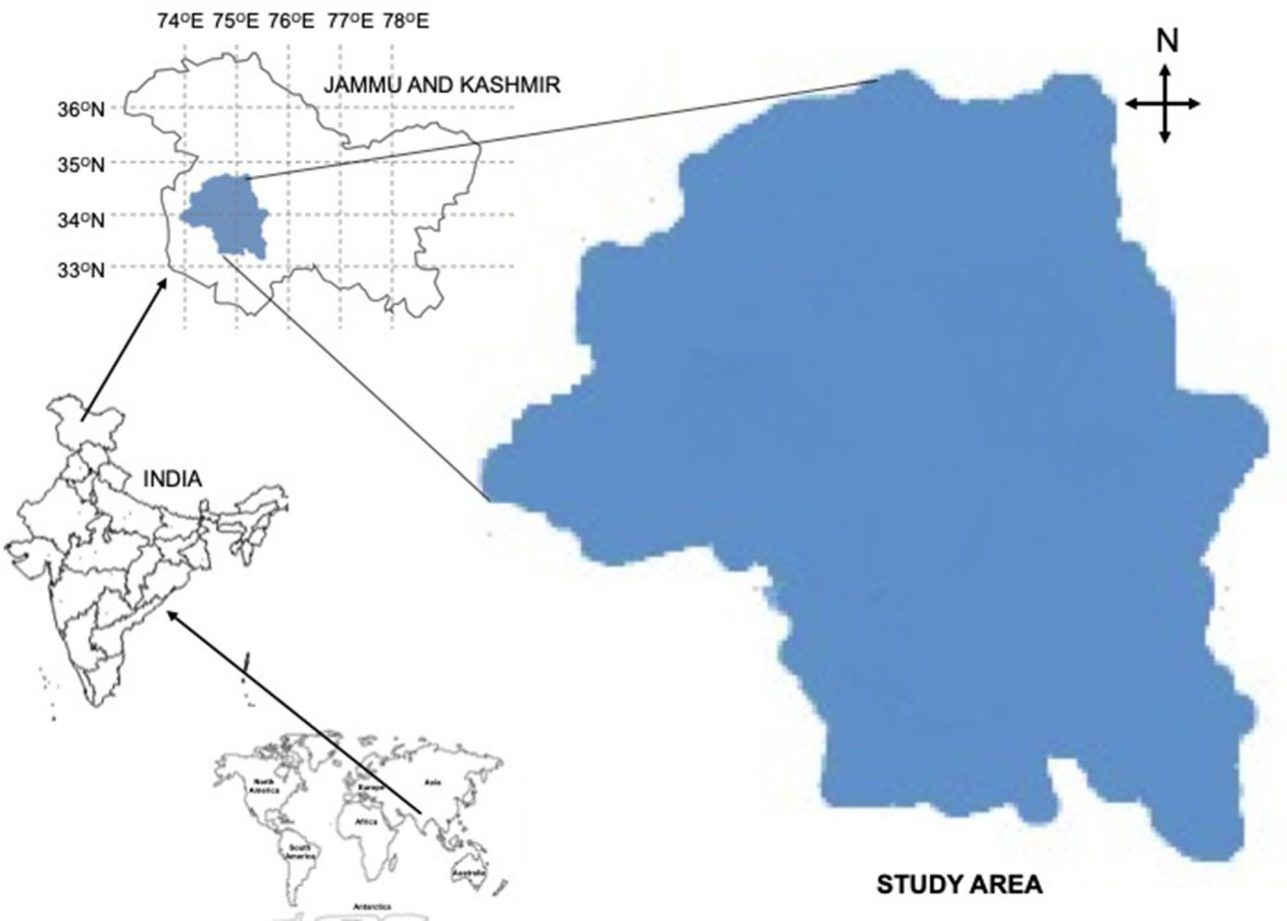
Location of the research/study area.

Life cycle of saffron is seven to ten years with varying yield in successive years^50^, and in present investigation an average of five years data was considered with the assumption that saffron yield within first five to six years is economically reasonable^26^. Geographically the study/concerned area extends from 32° 30′ to 34° 30′ N latitude and 74° to 75° 30′ E (Fig. 7). Agro climatically the region falls under temperate zone. The soils are slightly acidic with high organic carbon content (1.27%), medium available nitrogen (291 kg ha^−1^) and phosphorus (13.88 kg ha^−1^), and high available potassium (358 kg ha^−1^) status. Average available sulfur, calcium and magnesium are around 11, 994 and 260 mg kg^−1^. The infiltration rate of the soils is 16 mm h^−1^ and bulk density is 1.4 g cc^−1^. Texturally the soils are clay loam with electrical conductivity of 0.19 dS m^−1^.

### Experimental details

Present investigation was carried out through 5 year experimentation, from the year 2012 to 2017 with a set of thirteen N fertilizer placement/application methods (conventional as well as introduced), to reveal the impact of various modes of N fertilization on environment as well as yield of saffron. Thirteen N fertilization treatments were-Control (C), Broadcasting in one split (*conventional method I*) (B-1S), Broadcasting in two splits (*conventional method II*) (B-2S), Band placement single sided parallel to corm in one split (BPSSP-1S), Band placement single sided parallel to corm in two splits (BPSSP-2S), Band placement single sided upper to corm in one split (BPSSU-1S), Band placement single sided upper to corm in two splits (BPSSU-2S), Midrib placement parallel to corm in one split (MRPP-1S), Midrib placement parallel to corm in two splits (MPPP-2S), Midrib placement upper to corm in one split (MRPU-1S), Midrib placement upper to corm in two splits (MRPU-2S), Foliar application in one split (FA-1S) and Foliar application in two splits (FA-2S). The macro nutrients were applied as per recommended dose of N:P:K (90:100:120 kg ha^−1^)^51,52^ in all the treatments except in control, where no N was applied. In general, the saffron growers of the area either apply no nitrogen or recommended nitrogen (90 kg ha^−1^) through broadcasting. No nitrogen leads to decrease in yield and broadcasting leads to nitrogen losses, low nitrogen use efficiency and subsequently decrease in yield, although not as much as in cases where no nitrogen is applied.

Field experiment with these thirteen treatments was carried out at experimental site A having latitude 33° 58′ 54.58″ N, longitude 74° 48′ 3.49″ E and altitude 1640 m amsl, whereas the clay mineralogical and nitrate leaching studies were carried out at four different sites where conventional mode of N application was followed. All the treatments were replicated thrice in micro plots of size 6 sq m (3 m × 2 m) with randomized complete block design. The distance between the rows and corms was kept at 15 and 10 cm, respectively. The corms were sown at a depth of 15 cm in raised beds. Placement of fertilizers was done with exclusively designed V shaped single sided Pickaxe (*Tungroo*). The saffron corms have a characteristic of moving a little upward every year, so the placement was adjusted as per it. Nitrogen was applied through Urea.

### HPLC analysis for apocarotenoid quantification

All the saffron samples were dried under uniform conditions using saffron dryer to nullify the effect of drying conditions on the concentration of apocarotenoid^53,54^. For apocarotenoid analysis, extraction of saffron stigma was made with methanol (100 ml) in a micro-centrifuge tube for five minutes on ice, and then 100 ml of Tris–HCl was added and incubated for ten minutes on ice. After centrifugation at 3000*g* for 5 min at 4 °C, the precipitate was collected and the pellet was reground in 400 ml acetone and again incubated on ice for ten minutes. The mixture was again centrifuged at 3000*g* at 4 °C for five minutes. The process was repeated till the disappearance of colour of the pellet. After pooling and evaporation of the supernatants, the dried residues obtained were stored at − 80 °C until high performance liquid chromatography (HPLC) analysis. Samples extracted were then dissolved in HPCL grade methanol at a concentration of 100 mg L^−1^ and filtered through 0.2 µm syringe filters. Filtered sample (20 µl) was then injected in HPLC coupled to a photodiode array (PDA) detector^53^. With an injection volume of 20 μL and a flow rate of 1 ml min^−1^ with a run time of 35–40 min, the separation was done. Each sample was run in triplicate. Safranal and crocin were detected at wavelengths of 310 and 440 nm, respectively. The standards for respective apocarotenoid were also detected at the same wavelengths^55^. Chromatographic separation was performed on C18 (250 mm × 4.6 mm), 5 µm column using a solvent system comprising of 75% acetonitrile and 25% methanol in an isocratic mode. The mobile phase was filtered through a 0.45 μm membrane filter before analysis. Quantification of apocarotenoid was done by considering the respective peak areas of standards at particular retention time versus concentration and the values were expressed in mg g^−1^ of saffron stigmas.

### Gas collection and N_2_O analysis

The closed chamber technique was used to collect gas samples for N_2_O determination^56,57^. Air tight chambers, open from one end, of dimension 30 l × 30b × 90 h cm were made of 8 mm thick glass. These were inverted over hollow steel cubical channels (15 cm in height with 5 cm open cubic channel at the top, filled with water), and inserted 10 cm in the soil to make the system air tight. These chambers were installed in all the plots. Gas samples were obtained using a 50 ml syringe having a hypodermic needle through 3-way stopcock fitted over each syringe to make it airtight^58^. In order to measure N_2_O N fluxes, the volume of the chambers was recorded. Nitrous oxide N concentration in gas samples was analyzed by gas chromatography technique. Mean emission of N_2_O on the sampling days was interpolated linearly in succession to measure total N_2_O emission during the entire cropping season. A linear trend in N_2_O emission during the no sampling days was considered^22^. The gas sampling started during the first week of April, with fifteen day intervals, up to last week of November with the assumption that N_2_O emission during the remaining months is non-significant due to low temperature. Frequency of sampling was increased to four times in a fortnight during the events of N fertilization.

The global warming potential (GWP) on a 100-year time horizon was determined for all the modes of N fertilization using the equation developed by the Intergovernmental Panel on Climate Change (IPCC)^59^.

$${\text{Nitrous oxide GWP }}\left( {{\text{kg C}}{{\text{O}}_2}{\text{eq}}.{\text{ h}}{{\text{a}}^{ - 1}}} \right)\, = \,{{\text{N}}_2}{\text{O emission }}\left( {{\text{kg h}}{{\text{a}}^{ - 1}}} \right)*298.$$

(Where N_2_O emission (kg ha^−1^) = N_2_O–N (kg ha^−1^)*3.143).

### Leachate, runoff collection and nitrate analysis

Real soil column technique was used to collect leachate and runoff. The soil was dug from four sides in a square pattern and a square column with 14 cm side length was exposed. Glass chamber with sharp edges at the bottom, having ‘mm’ marking, was inverted over the exposed column and was pushed down gently with slow thumping. Because of the sharp edges (at the bottom) of the glass chamber, it goes gently downward and covers the whole exposed soil column. The soil column is cut from the bottom gently with least soil disturbance and taken to the lab for leaching and runoff studies. The glass chamber filled with soil column is put over a sieve, having a cone with filter paper in it. The cone opens in a Leachate collection flask. A running chamber of 2 cm thickness is attached to the glass column to collect the runoff. The running chamber is connected with a tube that opens in the runoff collection flask. The water is added to soils as per the recommended schedule (in mm) and fertilization (weight basis) is made as per the treatments. The leachate and runoff are collected in the flasks and the liquid is analyzed for NO_3_^-^ concentration by *Phenol Disulphonic Acid Method*^60^.

### XRD analysis and quantification of clay minerals

Clay minerals were studied using X-ray diffraction technique^61^. Quantification of clay minerals was done by taking into account the peak area of XRDs at different d-spacing.

### Nitrogen use, accumulation efficiency and statistical analysis

Nitrogen concentration in corms and the shoot was determined by Dumas total combustion method^62^. All plant tissue samples (leaf as well as corm samples) were washed, oven-dried, ground, and analyzed for Nitrogen by the Dumas total combustion method. Nitrogen use efficiency (NUE) was determined by using the formula.

NUE (%) = [{Nitrogen in shoot + corm (g kg^−1^) in treated plant—Nitrogen in control plant (g kg^−1^)} / Nitrogen applied (kg ha^−1^)] × 100.

Whereas Nitrogen accumulation efficiency (NAE) was determined by the formula.

NAE (%) = [Nitrogen in shoot + corm (g kg^−1^)/ Nitrogen applied (kg ha^−1^)] × 100.

Statistical analyses were done using SPSS 16 software and MS Excel.
